# On the Anatomy of the Breast

**Published:** 1840-07

**Authors:** 


### Art. V.

*On the Anatomy of the Breast.* By Sir Astley P. Cooper, Bart., f.r.s., d.c.l., g.c.h., Serjeant-Surgeon to the Queen, &c. &c.—*London*, 1840. 4to, pp. 265. With a Volume of Folio Plates.

It was with the utmost gratification that we saw the announcement of another work from the pen of Sir Astley Cooper; and great as was our anticipation of its merits, we must confess, after a careful and candid examination, that it has surpassed our expectations—by the importance and interest of its matter, the extent and accuracy of its details, the fidelity and elegance of its illustrations, and masterly style of its general execution. We may be permitted thus, in general terms, to express our admiration of the work before us; but we are well aware that praise in the abstract is far from being the proper form in which this meed should be awarded to Sir Astley Cooper; and assuredly this is not the mode in which we are accustomed to commemorate the deserts of distinguished men. Sir Astley Cooper belongs to that small class of historical personages, the simple enumeration of whose doings is the only eulogy worthy of them;

"And those who paint them truest praise them most."

Honestly to detail the motives which prompted his numerous investigations, the manner in which they were conducted, and the results to which they have led, is to claim and to obtain for Sir Astley Cooper the spontaneous admiration and willing homage of all who are qualified to judge of the achievements of scientific men.

But the medical profession is indebted to Sir Astley Cooper for much more than for the improvements he has effected in medical science. As a man and as a surgeon he is equally an honour to our time; and alike in studying his life as his works, we gather a stock of invaluable knowledge for the guidance of our conduct in our private and professional career.

And here it will be but justice to ourselves and readers—this being the first occasion since the commencement of our Journal that we have found an opportunity of calling attention to the subject—to take a brief survey of some of the principal improvements for which surgery and anatomy are indebted to Sir Astley Cooper. Many of the beneficial changes introduced by him were slow and gradual in their course, but to the eye of the future historian, as he regards them collectively, they will bear the character almost of a revolution.

Sir Astley Cooper was the first to ascertain with any degree of certainty the rate at which the ingesta are assimilated. So far back as 1792 he tied the thoracic duct of a dog, where it enters the vena innominata, and found that, under such circumstances, the receptaculum chyli burst three hours after a hearty meal; thus proving the rapidity of chylification, the great degree of contractile power possessed by the coats of the duct, and also, by inference, the fact that very few of the absorbents can terminate in the veins. In his observations on the defects in hearing which take place from the obstruction of the Eustachian tube, he was the first surgeon who sought scientifically to account for the derangements in the function of that organ; and though the operation of puncturing the membrana tympani, which he proposed in these cases, cannot now be considered infallible, it was still no less, at the time at which he wrote, a proof of his sagacity and of the soundness of his scientific principles. Spina bifida, a disease previously regarded as hopeless and intractable, he was the first to bring under successful medical treatment. By repeatedly puncturing the arachnoid, and removing the fluid which in this disease it incloses, he frequently caused it to take on a new and healthy action, and thus succeeded in suppressing the dropsical effusion, and in enabling the child to survive an affection under which it would have otherwise sunk. It was on physiology that his practice in this as well as in every other instance was based; and it is in the application of general physiological principles to the treatment of peculiar and apparently anomalous cases, it is in thus extending the rule of surgery to tracts which had previously bid defiance to its powers that we recognize the surgeon of genius.

Our limits will not allow us to do anything like justice to the vast improvement which Sir Astley Cooper has effected in our knowledge and in the treatment of hernia. Let the student, however, compare the confused and vague ideas which prevailed with respect to this disease in its different situations before his scalpel was devoted to its examination, with the correct and definite chart which writers on hernia are now enabled to sketch for their readers, and he will be able to form some judgment of the obligations this branch of surgical anatomy lies under to our author. Here, too, we do not find the practical improvements accidental or unconnected, but the result of industrious research and mature reflection. He was the first who showed that the abdominal muscles do not form the immediate internal parietes of the abdomen, but that there is a fascia everywhere present between them and the peritoneum. Scarpa and others had, it is true, spoken before of parts of this fascia, but in such a vague and indistinct manner that the information they afforded was anything but practically useful. Sir Astley was thus the first to demonstrate the proper outlets through which the herniæ pass; he could hence indicate exactly where it was that the division of the stricture must be performed, and he consequently diminished to a great extent the risk of wounding arteries.

No one before Sir A. Cooper is recorded to have tied the common carotid artery. After performing this operation, he was led to enquire into the extent to which, on the obstruction of main trunks, the circulation is capable of being continued by collateral branches. From experiments on the dog he found that the normal course of the blood admitted of such extensive modification, that even when the aorta was tied it could still be carried on. He was hence induced, in a case of aneurism of the common iliac, to tie the aorta in the human subject; the operation terminated unfavorably, but from extrinsic causes, so that no charge of rashness can, with a shadow of reason, be brought against him. There are numerous preparations in existence showing the spontaneous obliteration of the aorta, and there is certainly no reason why the operation in question should not succeed, where the general health of the patient is such as to admit of his bearing it. Sir Astley has tied all the principal arteries in the body, and has contributed much to the confidence with which the modern surgeon reckons on the establishment of collateral circulation when ligatures have been passed round main trunks.

Some idea of the value of Sir Astley's labours to improve the diagnosis and treatment of dislocations and fractures may be derived from the single fact that thirty-five years ago it was considered doubtful whether dislocation of the hip ever occurred: it was believed that if by chance it did take place its reduction was absolutely impossible. His is the earliest work on this subject in which the diagnostic marks of dislocations are systematically given. He was the first who insisted on the importance of constitutional means in reducing dislocations. Simplicity was the chief feature of the new treatment, of which the superior success soon became evident. Here we recognize, again, the scientific mind, of which it is the characteristic to produce the most striking and beautiful results by the fewest and simplest means. Formerly, in many cases of dislocation compounded with fracture, amputation was uniformly resorted to (for instance in that of the ankle-joint); but Sir Astley banished this practice in all but very bad cases, showing that nature could be trusted to repair the injury. Scientific observer as he has always been, and not a mere operator, he has never, with a rash and impertinent hand, officiously intermeddled with the natural processes of reproduction and repair. His system of practice affords a constant lesson to those who thrust themselves in the way of nature, and seek to figure by obstructing her remedial power. In compound fractures, Sir Astley never applied any other dressing than lint dipped in the blood, which he always left until it was loosened by suppuration. His apparatus of splinters, bandages, &c. was always of the simplest description. The extent to which his practice in this particular branch has been approved of and adopted may be judged of from the fact that his work on Dislocations and Fractures has passed through ten editions.

His Treatise on the Anatomy and Diseases of the Testis introduced light and order upon a subject where obscurity and uncertainty had previously prevailed. The perspicuity of his anatomical knowledge led to distinctness and decision in his surgical practice. Before his work was published, chronic abscesses, hematocele, and even hydrocele were not unfrequently removed from the testicle as malignant diseases; now such mistakes could only be committed by the wilfully ignorant.

It was the same spirit of scientific investigation which always suggested Sir Astley Cooper's labours, that prompted him to direct his attention to the nature and function of the thymus gland. He did not commence his studies in this department of anatomy by a theory; he patiently examined the structure of the part and was jealous, as he has ever been, of arriving at conclusions not strictly warranted by facts. The result of his researches was that the secretion of the thymus gland is highly albuminous and well fitted to renovate the venous blood. The general plan of anatomical and physiological enquiry adopted by him can be admirably studied in its application to this subject of minor practical importance, because the attention is not exclusively occupied by the magnitude of the questions at issue. His industry, his faithful adherence to nature, his minute yet comprehensive observation of phenomena, and the cautiousness of his advances towards a conclusion, are all admirably evidenced in the work on the thymus gland. His preparations, from which the statements in the book are derived, are not less than 250 in number. Indeed, had he not arrived at any valuable results the world would still have been much benefited by his having devoted his attention to this subject, as the truly scientific manner in which he has treated it cannot but be highly instructive as an example to all anatomical enquirers in these days of hasty speculation and loose research.

Sir Astley Cooper was one of the first surgeons who proposed the important improvement of cutting into the membranous portion of the urethra, instead of puncturing the bladder, in cases of retention of urine. —He has given to the world an excellent description of encysted tumours, which originate, according to him, in the obstruction and enlargement of cutaneous follicles.—In treating exostoses, he was the first who proposed to cut off the periosteum and thus cause them to die. We doubt also whether any surgeon before him recommended the operation of sawing them off. But we must here close our list of his improvements in surgery, which, were it complete, would comprise a reference, more or less extensive, to the treatment of the greater part of the affections which it is the province of that art to remedy. We cannot omit, however, to devote a few words to the general features of his practice.

Sir Astley Cooper has received from nature a happy mixture of boldness and caution, and it is the spirit thence derived which has guided him in the study of all diseases, and led him to improve or modify the old treatment, where it has not prompted him to have recourse to new measures. His genius has not been manifested in his brilliant discoveries alone. It has moulded his whole practice; and he has always been as philosophical in the rationale of his treatment of slight cases (from which nothing striking or novel was to be elicited), and as careful in the details of its execution, as in the performance of those more heroic feats which have carried his fame to the ends of the earth. His general influence on the practice of surgery in this country has perhaps been most evident in the great share he has had in establishing pure induction as the only sure means of a just diagnosis, and in introducing a simplicity of treatment in accordance with the processes of nature. Before his time, operations were too often merely frightful alternatives or hazardous compromises; and they were not seldom considered rather as a resource of despair than as a means of remedy; he always made them follow, as it were, in the natural course of treatment; he gave them a scientific character; and he moreover succeeded, in a great degree, in divesting them of their terrors by performing them unostentatiously, simply, confidently, and cheerfully, and thereby inspiring the patient with hope of relief, where previously resignation under misfortune had too often been all that could be expected from the sufferer.

As a teacher, Sir Astley Cooper has never been excelled by any English surgeon. His discoveries were no hidden treasures; no sooner had he made them than he hastened, with liberal enthusiasm and a winning affability, to diffuse a knowledge of them amongst a large class of pupils. The whole present generation of English surgeons may, indeed, be said to have studied directly or indirectly in his school; and, assuredly, no master was ever more beloved and honoured, or sent more enthusiastic and grateful followers abroad into the world to propagate his doctrines. The manner of Sir Astley towards his patients has been universally admired; and his kindness and attention as an hospital surgeon have been properly appreciated: we know, moreover, that in his relation to his private patients his character always showed to equal advantage, though this of course cannot be so well known to the public. He never spoke of a fee in his life. Where he received none he made it a rule to regard the case as one of distress, and was glad to perform an office of charity. "If we receive more than we expect," he has often said, "we return nothing; why, then, should we complain on receiving sometimes less than we deserve?"

The ardour and activity of this eminent man are qualities demanding our highest admiration. His love for science and zeal in her pursuits have never cooled from the time he first entered the profession. Even at the period of his life when his days were unceasingly occupied by the demands of his public and private practice, he would spend the greatest part of many of his nights in dissection; and it was then well known that he was ready at any hour to obey a summons from any quarter to attend a post-mortem examination. Even now, at an age when nature usually diminishes if she does not entirely destroy the power of the understanding to exercise its highest faculties, we find him still proceeding in the same course, working incessantly, and ever and anon giving to the world some elaborate work, displaying all the zeal and lively interest in the subject, which are commonly regarded as the exclusive attributes of youth. Instead of sinking under the shadow of his fame into luxurious repose, or forgetfulness of his past exertions, the honours with which he has been crowned only seem to stimulate him the more to pursue vigorously in old age the same path which he entered upon so ardently in his youth.

In the prosecution of his enquiries, Sir Astley Cooper never broached crude opinions, nor indulged in idle theories. He has preparations in his own house confirmatory of all the statements he has made in his anatomical works; and these constitute perhaps the most splendid anatomical museum possessed by any private individual in Europe. His improvements in surgery have always been based on the result of his anatomical enquiries; and with respect to the latter, not content with the observation of particular facts, as too many are, he has never given his discoveries to the world except in a complete and satisfactory form. In preparing his last work, he has restricted himself, he tells us, to describing from his own preparations. "My rule," he says, "has been always to publish that only which I could show to those who were sceptical and were yet desirous of arriving at the truth." It is this self-evident truthfulness both as to statements and inferences, conspicuous in every page, that makes so very valuable all the writings of Sir Astley Cooper; it is from this that they all derive a worth which time cannot destroy, and which will always entitle them to be ranked among the noblest monuments of the epoch at which they were produced.

We must now proceed to the immediate subject of this article, and give some account of Sir Astley's latest work, on the Anatomy of the Breast. This was undertaken, like almost all his other works, to pave the way for improvements in the practice of surgery. Rather more than ten years ago he gave to the world a volume on the non-malignant Diseases of the Breast. It contained, among other things, an admirable diagnosis of the affections springing from common inflammation in contradistinction to those of a malignant origin, which he had felt to be much wanted, inasmuch as hydatids, for instance, had been frequently mistaken for fungus hæmatodes; and other errors of a similar kind had been far from uncommon. In this work he also showed what were the particular tumours which might be removed without fear of their returning. He at first intended to follow up this work by a second part, which was to comprise the malignant diseases of the breast. He found, however, that the anatomy of the organ had not yet been sufficiently investigated, or, at any rate, that very indefinite and incorrect ideas were still prevalent with respect to its structure; and hence, in accordance with the rule which has guided him in all his scientific investigations, he set himself to demonstrate the precise anatomy of the breast, before entering upon the pathology and treatment of its diseases.

The first of the two volumes before us is divided into twenty-four chapters or sections (for the divisions have no particular title), and contains besides an appendix of comparative anatomy, and descriptions of the plates, which constitute exclusively the second volume. The first chapter contains general observations and definitions, and an account of the mode in which the author's investigations were pursued. Chapters ii. to xi., inclusive, comprise the organization of the various structures of the breast in the human female. Chapter xii. describes the effects of gestation and the development of the breast, and comprehends a chemical analysis of the fluid secretion. Chapters xiii. and xiv. are devoted to the function of lactation, and to the changes produced in the breast by the protraction or frequent repetition of this process, by age and other causes. Chapters xv. to xxv. treat of the several structures and of the organization of the mammary gland in the human male.

The "cunning of excelling nature," in providing, in the breasts, an apparatus for the supply of nourishment to the young animal, so soon as it shall be separated from its parent, is universally known and admired. "Soon after the commencement of utero-gestation, they begin to receive an additional quantity of blood to prepare for the new secretion, and thus by an admirable foresight, when the link which united the offspring to the mother is broken, a new and entirely different mode of nutrition is substituted for that which it had previously received." (p. 1.) In describing the adaptation of the structure of the mammæ to their intended purposes, the author divides the parts of which they are composed into internal and external. The former, which constitute the glandular or secretory organ, are concealed under the skin; the external part, called the mammella, nipple or teat, is formed to convey the secretion of the gland to the offspring. "The former would be of no use without the latter, for, however abundant the secretion of milk, the infant would be unable to receive it, if the nipple or mammella had not been added to the breast or mammæ."

"In the human female the breasts are placed upon the anterior and lateral parts of the chest, in what may be called the mammary region; and here the child when sucking is placed immediately under its mother's eye, as it receives the nourishment from her breast. Here it almost irresistably solicits her tender and regular attention, and the demonstrations of that affection which ought to be in future life reciprocal between the parent and the offspring." (p. 2.)

Here we have not only the physiological expression of the facts but also their moral interpretation, helping in some degree to explain the characteristic distinction between the lasting, mutual affection which nature has ordained should unite the human mother to her offspring, and the merely instinctive attachment of the females of brute animals to their progeny, which ceases concomitantly with lactation. In proceeding to point out the different position of the mammary glands in different animals, the author directs our attention to the care displayed by nature in providing ready access to them for the young, and in protecting them from external injury during the motions of the animal. The size and number of the mammary glands, as well as the quantity of the secretion which they produce in different animals, bear no constant relation to the size or number of their offspring; the gland in the cow, for instance, is much larger in proportion than in the mare: then again the cow has four perfect glands and the guinea-pig two only, though the former animal has generally but one young, whilst the latter has several at a birth. It is very true that the guinea-pig, though she has frequent litters, and often nine or ten young at a time, is only furnished with two teats; but still it is not to be laid down as a general rule that the number of glands is not generally in proportion to the number of the offspring. The instance just quoted is rather, we think, to be regarded as an exception than as a law. In the bitch, cat, doe, sow, and many other animals, we observe that the number of teats is in proportion to the number of young, and must therefore conclude that this arrangement is the one most in accordance with the general design of nature. The author leaves the question open, merely remarking that the number of the glands does not always correspond to that of the offspring.

Notwithstanding these diversities, however, in the external character of the mammary glands, the organization of their secretory portion is very similar in all animals: "however complicated they may at first sight appear, their intimate internal structure really exhibits a remarkable degree of simplicity." (p. 4.)

The variation in the quantity of secretion from these glands in different animals is very remarkable; its abundance in the cow and goat, the author particularly alludes to, as showing a wise provision of nature not only for the support of their own young but for supplying man with the most wholesome of food.

The sources from which the mammæ derive their supply of blood vary according to the position of these glands in different animals; and even in the human subject, the source and course of the arteries are by no means uniform; but diversities of this kind do not interfere with the quality or quantity of the secretion. In this respect, it is observed, the glands in question differ from others, for example from the testis, to the performance of the functions of which the distant origin of the spermatic artery and the tortuosity of its course appear to be essential. The mammary veins constantly follow the varieties in the origin and course of the arteries. The nerves of the mammæ arise in all animals from the spinal marrow and sympathetic system, and from the cervical, dorsal, or lumbar portions, according to the position of the glands. The author concludes his first chapter by detailing the best method of exploring the various tissues of the breast, and of demonstrating and preserving each particular structure. For a clear and elaborate account of these difficult and delicate manipulations we must refer such of our readers as require instruction on this branch of the subject to the book itself, since the description does not admit of being curtailed, and is too long to be quoted in full.

The second chapter commences with an account of the proper anatomical position of the breasts, including a description of the region of the thorax on which they are placed, and a notice of the muscles on which they immediately rest. They are rather concave posteriorly in accordance with the convex surface of the thorax. The arched form of the ribs adds to their projection and facilitates the access of the child to the nipple. As external parts of the breasts the author enumerates the nipple, areola, tubercles, and mammary glands. The internal or the true secretory portion is composed of "an assemblage of small secreting bodies or granules, from which proceed the lactiferous tubes to the nipple." These granules and tubes are all bound together by a firm, fibrous, inelastic membrane, which, though on the one hand it may be described as connecting the various internal structures, may also be said, on the other hand, to insulate the different portions so as to make, on the whole, a conglomerate gland. A considerable quantity of cellular tissue also enters into the composition of the breasts, containing in different individuals more or less fat, to which the human mammæ are indebted for their smooth rotundity, and which also perhaps effects some more important object. The author indicates the manner in which the mammæ are attached to the thorax by stating that they are "*slung* upon the chest," and are so supported by the fibrous tissue already spoken of, that the nipples are projected forwards and outwards. He here complains that modern artists have not in their delineation of the human figure paid sufficient attention to the disposition of these parts, but have represented the nipple as pointing directly forwards. The ancient sculptors, he observes, modelled from the living subject, and therefore did not fall into this mistake. We cannot refrain from quoting the author's beautiful description of the object gained by the eversion of the nipple.

"This natural obliquity of the mammella or nipple forwards and outwards with a slight turn of the nipple upwards is one of the most beautiful provisions in nature both for the mother and the child. To the mother, because the child rests upon her arm and lap in the most convenient position for sucking, for if the nipple and breast had projected directly forwards the child must have been supported before her in the mother's hands in a most inconvenient and fatiguing position, instead of its reclining upon her side and arm. But it is wisely provided by nature that when the child reposes upon its mother's arm, it has its mouth directly applied to the nipple which is turned outwards to receive it, whilst the lower part of the breast forms a cushion upon which the cheek of the infant tranquilly reposes. Thus it is, as we have always to admire the simplicity, the beauty, and the utility of those deviations of form in the construction of the body, which the imagination of man would lead him à priori to believe most symmetrical, natural, and convenient." (p. 12.)

The position of the breasts prior to impregnation is much altered by frequent lactation, by the debilitating effects of a hot climate, and by exposure to a high degree of artificial warmth; but as relaxation invariably accompanies such changes, the infant is not prevented from sucking in the usual manner. Buffon, in his *Histoire Naturelle*, states that a line drawn from one nipple to the other, before any change has taken place in the natural position of the mammæ, would form the base of an equilateral triangle, of which the lines meet in the centre of the pit above the sternum. Sir Astley, however, informs us that in the Venus de Medicis the distance from one nipple to the other is $7\frac{5}{8}$ inches, whilst the pit between the clavicles is only $6\frac{1}{2}$ inches from each nipple, so that in this beau-ideal of female beauty the base of the triangle is longer than its sides. It is an important fact that the glandular portion of the breast frequently projects into the surrounding fibrous and cellular tissues, so as to destroy the regular form of its disc: this should be borne in mind in all surgical operations on the part and whilst removing it for anatomical investigation; or else, as our author observes, portions of the glandular structure will be almost certain to be left behind, leading, probably, in the former instance to a return of the disease for which the operation was undertaken, and rendering, in the latter, the removed organ quite unfit for injection and preservation.

We perfectly agree with the author that the human female is provided with two breasts, not for the purpose of enabling her to nourish twins, as is generally supposed, but that she may be capable of affording the necessary supply of milk to her offspring, should one of the mammæ be destroyed either by accident or disease. The comparatively rare occurrence of twin-births is in favour of this opinion, and still more the fact that women with supernumerary mammæ or nipples have not been found to be more prolific than is usually the case. Cases in which more than two breasts have existed are mentioned as occasionally occurring; but the author confines himself here to quoting one example of this anomaly (a woman with four breasts,) which he witnessed through the kindness of Dr. Robert Lee, in a letter from whom its history is given. The second chapter concludes with an account of the physiological and pathological causes of the varieties observed in the thickness of different portions of the mammæ. The axillary and abdominal edges are naturally more compact and dense than the sternal and clavicular portions.

"In this way the lower part of the breasts forms the cushion upon which the cheek of the child reposes as it seeks its mother's bosom; and as to the causes by which the greater thickness and projection are produced, I shall particularly point them out in speaking of the gland, but I may here observe that upon this structure depends the projection of the nipple, the ready access which the child has to it, and thus two important objects are accomplished." (p. 19.)

As the consistence of the substance of the mammæ is very various at different periods of life, being equally compact in the virgin, comparatively knotty and irregular during and sometime after lactation, and moreover being periodically affected by menstruation, it behoves the surgeon to render himself perfectly familiar with these natural changes, or he will be liable to confound them with morbid phenomena. He ought also to have an exact knowledge of the changes which the mamma undergoes from age. So soon as the period of menstruation ceases, an alteration, as might be expected, takes place in this organ, an apparatus for the secretion of the milk being now no longer required. Although the mammary glands themselves waste rapidly away at this time of life, their ducts still remain open for a considerable period, during which they are found loaded with a mucous secretion which may be squeezed from the nipple. Sir Astley Cooper sent some of this secretion to Dr. Prout, who found it to consist of phosphate and carbonate of lime. The distension of the ducts with this fluid is rather to be attributed to the difficulty with which it escapes through the contracted orifices of the nipple than to its being secreted in great abundance: during its retention the more watery parts evaporate, and thus the residue is inspissated. The milk-cellules of the breast in old age do not admit of being filled with injection, and the ducts even can then be only imperfectly penetrated. The most singular, though an almost invariable, change which age induces in this structure is the tendency of its arteries to ossify and to become impervious from the quantity of earthy matter deposited within them. The veins of the breast are much diminished by age, which, however, has no appreciable effect upon its nerves. As life declines the nipple becomes long, wrinkled, and relaxed, and in very old age it contracts and resembles only a warty excrescence. "It appears, then, that the effect of age is to absorb the glandular structure, to load the ducts with mucus, to obliterate the milk-cells, to ossify the arteries, to wrinkle and elongate the nipple, and at length in a great measure to absorb it." These changes are well delineated in plate viii. figs. 3, 8, and 9.

The third chapter or section of the work is confined to a description "of the structure of the constituent parts of the breast" in the female; but it is our intention while treating of these to compare them with the corresponding structures in the mammary glands of the male described in sections xv. and xvi. A juxta-position of these organs as they exist in the two sexes will be found to elucidate each other in a physiological point of view. In describing the particular breasts, however, we cannot improve upon the plan of the author, and shall always, like him, proceed from without inwards, beginning of course with the nipple.

The mammary gland of the male is but a miniature picture of that of women. Its size varies much in different individuals, and it appears, in the author's opinion, to be most developed in those who have an effeminate aspect. The largest he had ever an opportunity of observing were in a man whose testes were imperfectly developed. This coincidence, to call it nothing more, appeared to him so extraordinary that he has given a delineation of both the testes and mammæ of this individual in plate ii.

Physiologists have hitherto given but little satisfactory information with respect to the functional purposes of the mammary glands in the male. Whilst some authors seem to regard them as merely ornamental appendages to the frame, others go so far as to assert they are designed to provide nourishment for the young, as if nature had intended that the father should assist the mother in this important duty. In a letter to Sir A. Cooper from his nephew, Dr. Young, of Barbadoes, the latter says, "I have seen some cases of enlarged and pendulous mammæ in the negro bearing many of the external characters of those of the negress who had never borne children." Sir Astley's own opinion on this subject is as follows:

"It is true that from the mammary gland a very small quantity of fluid may be sometimes pressed through the nipple, and the continual application of the infant's lips might slightly increase the quantity of the secretion, and the child might be gratified by sucking the nipple as it is by sucking the finger, but the quantity of secretion is too small for the purpose of affording nutrition to the infant. It appears to me that its use is to form an organ of sympathy with the other parts of the sexual system, which are influenced and excited by mental impressions and by the direct irritation of the nipple. For this purpose the organ possesses an erectile tissue of arteries and veins, and a high sensibility from several nerves which are devoted to the supply of the nipple and mammary gland." (p. 161.)

We believe, however, though our author does not go into the subject, that under certain circumstances the gland in the male may be made to perform, to a certain extent, the office of the female breast. Humboldt mentions a man in the village of Avena, who, on the death of his wife, suckled her infant for five months; and other similar instances are extant, which certainly appear to authorize the belief that some degree of nourishment may, under certain circumstances, be derived from the male breast. This may not be the specific object of the organ in the male, but it would still be unphilosophical to conclude that it is impossible for the male breast to become, in some degree, a substitute for the breast of the mother. It seems to be also authenticated that girls before puberty, unimpregnated women, and even females advanced in age are capable of furnishing support to a child, if their breasts have been frequently stimulated by the application of its lips in the attempt to suck. This proves that an increased flow of blood, induced from other causes than sympathy with the uterus, is able to produce a nutritious secretion. Nor is such a result at all anomalous, for we may generally expect where there is a determination of blood to any secretory gland, that there will be an increase of its secretion, not so perfect, perhaps, as that proceeding from the appropriate stimulus, but still of a very similar character. Of the great sympathy and connexion between the mammary glands and the other parts of the sexual system in the male we are quite convinced, having met with several cases in which it was manifested, and with one in particular, that of a friend, who has seriously avowed to us that the slightest irritation of his nipple invariably excited in him sexual desire.

On considering the whole organization of the mammary glands in the male as they are elaborately described, especially with respect to their absorbent system, by our author, we cannot but think that they have other functions than those which have been hitherto noticed. With respect to their larger size, however, in many individuals, this is generally owing more to an accumulation of fat than to the magnitude of the gland itself. The author has seen them very large in an old negro, and seems inclined to believe that they increase in proportion to the decline of virility in man, which, if it be really the case, is the more remarkable, as in the female they begin to shrink and diminish from the period when menstruation ceases.

In considering the particular structures which constitute the male and female breasts, we find a wonderful similarity to exist between them in the two sexes. We now proceed to notice the different parts.

*The nipple or mamilla.* The nipple of the male resembles so strongly that of the female in position, form, and physical condition generally, that in describing the one, under these heads, we should also be giving an account of the other; but in respect to function the mamilla of the female is so much the more important that we shall devote our attention principally to it. The female nipple is most carefully described by the author with the intention of showing that it is situated in the best possible manner for conveying the milk from the breast of the mother into the mouth of the child.

"It springs from the convex surface of the breast, and projects forwards and outwards, the point being also generally directed slightly upwards.....It is not placed in the centre of the breast, but is situated nearer the abdominal margin of the gland than the clavicular edge.....It is placed below a line drawn across the middle of the gland, from the sternum to the axilla, and on the outer side of a vertical line drawn from the middle of the clavicle to the abdomen.... In the female infant it is placed upon the edge of the fourth rib. At puberty it descends to between the fourth and fifth ribs, but, after several lactations, it often descends as low as the seventh rib." (p. 22, 23.)

The form of the female nipple is that of a cone, rather rounded at its extremity in the virgin, but becoming flattened during lactation, in consequence of the unfolding of the cleft which had hitherto concealed the terminations of the lactiferous tubes. This change of form renders the adhesion of the child's lips much more firm and complete. The male nipple remains rounded and but slightly elevated through every period of life.

The colour of the female nipple depends to a great extent on the condition of the uterus at different periods. "In infancy it is of a pinkish red; at puberty of a more florid red; in young women of a slight brownish red; but in pregnancy it becomes of a very dark colour. In old age it becomes again more of the hue of the surrounding skin, although sometimes it remains very dark." The author does not notice any analogous changes in the male nipple corresponding to the various conditions of the organs of generation in the successive stages of life.

The nipple, both in the female and male, is composed of the following structures: common integuments, fascia of the ducts, ducts, arteries, veins, absorbents, nerves, with a general investing cellular membrane.

The cuticle of the nipple in both sexes, though resembling generally that of the rest of the body, may perhaps be said at its apex to offer a greater resistance to separation from the subjacent structure than usual; but this results not only from the irregularity of the surface of the nipple, but also from the connexion of the cuticle with the mucous membrane of the lactiferous tubes, which is rendered evident when it is removed by maceration or decomposition, by one or other of which processes the tubes themselves can be best demonstrated. Our author remarks that the tenuity of the cuticle in some females occasionally leads to such great inconvenience during lactation, that the application of astringents or mechanical protection is required. We have known serious affections to arise from a similar delicacy of cuticle in the male, leading, in some cases, to a protracted irritable condition of the mammary gland, and in others to the formation of abscess, though the exciting cause had been sometimes nothing more serious than the mere friction of a brace.

The rete mucosum of the human nipple presents no peculiarities. Into the organization of the cutis of the nipple our author enters at too great a length for us to be able to follow him; but we shall endeavour to lay before our readers a succinct account of his views with respect to its functions. The cutis of the circumference of the nipple differs considerably from that of its truncated apex: the former is remarkable for its numerous papillæ, rendering it highly sentient, whilst the latter is pierced by the orifices of the lactiferous tubes: this diversity is of course most obvious during the time of lactation. The direction of the papillæ just mentioned is from the base towards the apex of the nipple, so that they are pushed back as the mamilla enters the mouth of the infant, and thus produce the excitement necessary for the secretion of milk. Their foliated form and vascularity are beautifully delineated in plate ii. figs. 12, 13, 14, 15.

In treating of the vascularity of the nipple, Sir Astley has described the termination of the arteries in the veins as it takes place in each papilla; and he insists particularly (in a note, p. 28,) that the anastomosis of arteries with veins is direct, and that the communication between them is not effected by any intermediate vessels, as is now generally maintained. The erection of the nipple, which is so evident during the process of suckling, has been supposed to arise from the passage of blood into a structure similar to the corpus cavernosum penis; but the author attributes its distension simply to a determination of blood into the capillaries of the nipple and papillæ.

The openings of the lactiferous tubes, which, except during lactation, are inclosed in the cleft dividing the apex of the nipple, are extremely minute, both in the human female and in other animals, so as to prevent the escape of the milk, to aid in retaining which there is no transverse wrinkling of the tubes, as Haller states: "but as any one may at once see, by cutting them open near their terminations, they are wrinkled longitudinally, to allow of a greater dilatation of the tube behind the contracted orifice."

The nipple is attached to the mammary gland by means of the lactiferous tubes, the suspensory fibrous tissue, and cellular membrane, which together prevent its too great elongation and relaxation, and preclude the possibility of its being separated from the breast by external violence, whilst at the same time they are sufficiently flexible to admit of its very various changes of position, to suit the motions of the child whilst suckling. The delineation of the arteries, veins, nerves, and absorbents of the nipple (plate ii. fig. 14, 15,) display at once the minuteness and accuracy of the author's anatomical investigations.

There are four principal arteries of the nipple, derived from the axillary and internal mammary arteries; both sets pass to the basis of the nipple, where they freely inosculate, and send parallel branches to the apex and papillæ, whilst others pass backwards to the lactiferous tubes and communicate in the centre of the gland with branches from the intercostals. The veins of the nipple, at their origin, form bundles or bushes, proceed in larger trunks to the venous circle of the areola, and ultimately terminate in the axillary, internal mammary, and intercostal veins.

There are two sets of nerves, a posterior or axillary, and an anterior or sternal. The former, derived principally from the fourth and fifth dorsal or intercostal nerves, proceed to the nipple, supported on branches of arteries; the latter consist principally of the reflected branch of the fourth dorsal.

With respect to the absorbents of the nipple, which we ourselves, in common we believe with many other anatomists, have never seen, we cannot do better than quote Sir Astley's own description:

"The absorbents of the nipple, which are very large and numerous, proceed from its basis along the surface of the gland to the axillary fascia, where they pass through its cribriform absorbent opening or openings to terminate in the axillary absorbent glands immediately behind the fascial aperture, and a little above it, close to the edge of the pectoralis major. But the absorbents on the sternal side of the nipple take two courses into the anterior mediastinum, viz. between the cartilages of the second and third ribs, and between the fourth and fifth." (p. 34.)

*The Areola.* The fourth section or chapter is devoted to the Areola. It is nearly on a level with the common integuments of the breast. The nipple springs out from its centre before lactation, but below the centre in lactating women. The diameter of the areola is only half an inch in the infant, and an inch at puberty; but during lactation it frequently extends beyond two inches; before which period both it and the mammary gland are in what may be termed a rudimentary state. Its colour varies as life advances, corresponding in its changes to those in the tint of the nipple already described. The change of colour in the areola during pregnancy the author dwells upon as diagnostic, and as hence important both to the medical man and to the female herself, but he does not consider it peculiar to that state, and therefore not an invariable criterion, having seen it exist under certain exciting conditions of the uterus. Its cuticle, like that of the nipple, is delicately attenuated, that it may not interfere with the sensitiveness of the cuticular papillæ: in some cases this tenuity is so extreme as to lead, as in the nipple, to inconvenience during suckling. The rete mucosum of the areola is subject to various changes of hue according to the different states of the uterus. The immediate cause of these changes the author seems to attribute to irregularity in its supplies of blood. Thus, during pregnancy, a greater quantity of blood being sent to the breast, the rete mucosum assumes its deepest shade, while in the decline of life it regains its general complexion. Somewhat analogous are the effects of a hot climate, which we constantly see affecting the complexion. We do not quite agree with the author that the reticulated character of the rete mucosum is derived from the form of the inner side of the cuticle, but are rather disposed to believe that it is attributable to the existence of a congeries of veins anastomosing at right angles. A similar venous network is found generally under the cuticle, and is very evident in the negro: and it is owing to this arrangement probably that the skin is able to eliminate such large quantities of carbon, and thus to assist in the function of the lungs. The areolar cutis is plentifully supplied with papillæ, which are rendered very evident by the separation of the cuticle and rete mucosum. They are stated to be smaller than those of the nipple, towards the base of which, however, they increase in size. They are highly vascular and sensitive, and serve to increase the adhesion of the infant's lips in suckling: they also add to the sympathy of the areola with the mammary gland. The areola is to be regarded as little else than an extension of the nipple, with which its organization is nearly identical.

The tubercles of the areola have never, as far as we have been able to ascertain, been properly described by any previous writer on the breast. Sir Astley has succeeded even in injecting them, and demonstrating their glandular structure: they are beautifully delineated in his third plate. Their orifices, placed at the circumference of the areola, vary in number. They are described as highly vascular and lobulated, and as serving to lubricate, by their secretion, the surface of the areola, to add firmness to the adhesion of the child's lips, and to give greater sensibility to the areola. We cannot well understand why the author should attribute sensibility to this secretory apparatus; we think that a second definition which he gives (at p. 45) of their functions is more correct, where he says that they are merely mucous glands formed to lubricate and defend by their secretion the nipple and areola. They increase considerably in size during lactation. Morgagni, Meckel, and others, have thought that their orifices communicated with the lactiferous tubes, and that milk could be squeezed out through them: the fallacy of this opinion Sir Astley has clearly proved. The skin around the areola, and indeed of the whole breast, is particularly smooth, firm, and white, owing, Sir Astley says, to the intermixture of the glistening fibres of the fascia with the cutis. On minute examination, a number of small glands may be discovered on the skin of the breast, which serve to moisten its surface: their orifices often contain fine hairs: these glands are beautifully delineated. (pl. iii. fig. 5.)

The structures of the male areola are precisely the same as those of the female, and in infancy its organization in the two sexes appears identical. It undergoes a change at puberty, both in the male and female, and now its higher importance in the latter becomes evident from its more rapid development: in the male after puberty its increase in size is only proportional to the general growth of the body. With respect to the origin and course of its vessels and nerves, the male nipple does not differ from that of the female. On the function of its absorbents we shall defer our observations till we have described the internal structures of the breast.

In the fifth section the description of the internal structures of the breast is entered upon: viz. the fascia mammæ, the lactiferous tubes, the lacteal glandules, the milk-cells, the organization of arteries, veins, absorbents, and nerves, and finally the fat and cellular tissue.

*The Fascia of the Breast.* To Sir Astley Cooper belongs the merit of having first pointed out the striking part which the fascia mammæ performs in the economy of the breast. He describes it as consisting of two layers, which, at the sternum, adhere to the ligamentous substance covering that bone. Thence they proceed outwards, separate from each other, and inclose the breast between them. At the axillary margin they again unite, and, becoming fixed to the fascia of the axilla, the breast is "slung" in a fibrous covering. The anterior or superficial layer is firmly united to the integuments of the breast by numerous processes termed by our author *ligamenta suspensoria,* between which lobes of fat are deposited, giving rotundity to the organ, and serving to defend it from external injury. From the posterior surface of this anterior layer numerous processes pass backwards into the substance of the mammary gland, intersecting and connecting its various portions. A process of this fascia also proceeds to the nipple, surrounding and inclosing the ducts which are contained within it. The posterior layer of the fascia mammæ also sends fibres into the substance of the breast, whilst others pass backwards, and are united to the aponeurosis of the pectoralis major. After this excellent description of the anatomy of the fascia mammæ, its important uses will suggest themselves at once. Its arrangement is admirably demonstrated in fig. 1, 2, 3 of pl. iv.

*Excretory Ducts.* In treating of the secretory structures, we shall deviate somewhat from the arrangement of the author, and commence with the excretory ducts, the orifices of from seven to twenty-two of which are found in the cleft of the nipple. These orifices are not uniform in size, but are without exception narrower than any other portion of the tube, as if for the purpose of preventing the escape of milk. On tracing the ducts backwards, they will be found to dilate considerably as they approach the areola, where they open into large branches, formed by the union of several lactiferous tubes from the glands; thus we find that there is a reservoir for the milk between the secretory or internal, and the excretory or external portions of the breast. The mammary, lactiferous or milk tubes arise in small and numerous branches from the glandules of the lobes: if an injection be thrown into a tube formed by the united branches of one lobe it will enter that lobe only, and will pass into no other. In pl. vi. Sir Astley has exhibited drawings of numerous lobes of the breast, injected with various colouring matters, and without any blending of the different tints. The interior of all the excretory ducts of the human breast is lined by a highly vascular mucous membrane.

*The mammary Gland.* The gland of the breast is constituted by a number of glandules, connected by processes of the above-mentioned fascia mammæ. Each lobe may be unravelled into lobules, and each lobule again into glandules; and the whole may thus be made to present the appearance of a bunch of currants hanging from its stalk. The size of the respective parts depends upon the period of life, and on the condition of the mamma itself in reference to the state of the uterus. In the unmarried female, after puberty, the several parts may be demonstrated, but they are small; they enlarge however somewhat during menstruation. During lactation they are large, and can be easily demonstrated. As life advances the glands gradually diminish, but the tubes do not alter much. One of the great uses of the attachment of the glands to the skin by the "ligamenta suspensoria," is to render the breast more prominent, and the nipple consequently easier of access to the lips of the infant. The numerous folds which the fascia presents for the reception of the glandules also afford an immense increase of surface for secretion, without increasing inordinately the size of the breast. The disposition of the fascia with regard to the glandules is rendered very obvious in pl. vii. fig. 2.

The glandular structure of the axillary and abdominal margins is much thicker than that of the sternal and clavicular margins, owing probably to its being folded in the former on itself, so as to form a cushion for the child's head, where alone this can contribute by its pressure to aid during suckling in the expulsion of the milk. At the lower and outer part of the mamma there is a greater number of granules and of excretory ducts than in any other situation, and the lactiferous tubes are here placed one upon the other. The posterior surface of the mammary gland is not so irregular as the anterior, from its not being disposed in folds or plates in the same manner by the fascia. The circumference of the breast is not an exact circle from some parts projecting further than others into the subcutaneous tissues.

The milk cells are found in the interior of the granules, and differ in number according to the size of the latter. They are lined by a membrane which Sir Astley seems inclined to denominate serous, though he acknowledges that its vascularity renders it more similar to the mucous membranes, to which class we think there can be no doubt that it belongs, judging both from analogy, from its anatomical character, and from the circumstance that were it serous the first inflammatory attack would at once obliterate the cell and destroy its function. The arteries which supply the cells with blood secrete also the milk : their terminations are extremely minute; and although, at the period of lactation, the trunks themselves increase considerably in size, no corresponding change occurs in the capillaries. The veins convey the refluent unexpended blood into the general circulation. The absorbents connected with the milk-cells are very numerous, and perform the double function "of absorbing the more watery particles of the milk so as to render it more nutrient than under its first secretion; and are also employed under great accumulations, in the absence of the child, when they relieve and unload the vessels." There are, however, cases where, notwithstanding the operation of the absorbents, recourse must be had to the lancet for the relief of accumulation, as the author has shown in his work on the Non-malignant Diseases of the Breast. The nerves of the secretory structure are extremely minute, and take the course of the arteries on which they are supported. Sir Astley concludes this section with so happy a description of the secretion and course of the milk that we should not be doing justice either to him or to our readers were we not to quote it:

"From this description of the structure of the parts the function of the organ is easily explained. The milk is secreted by the arteries into the milk cells, from which it is forced forward by two causes; first by the elasticity of the cells, which is proved to exist in many animals by injecting the cells minutely with quicksilver, and then if one of the ducts be pricked with a needle all the lactiferous tubes become instantly emptied, but in women this occurs less than in other animals. Secondly, by the vis a tergo of the continued secretion, one portion of milk forcing forward the other, in a minor degree when the child is not applied, but when the draught occurs a sudden rush of blood increases the secretion, and rapidly hurries the blood forwards to the nipple, to supply the wants of the infant. The milk is conveyed from the cells which are found in every point of the gland into the *mammary ducts,* which form radii converging all of them towards the areola; and, as these vessels are increasing in their diameters, little opposition is made to the progress of the milk as it courses from the smaller to the larger tubes. When the milk is thus brought by the mammary tubes to the areola, it is received into the *recevoirs,* and in these, and in the mammary ducts, it is retained until the infant begins to suck; and here it will be seen that the form of the tubes is reversed, for the mammary tubes are constantly increasing towards the nipple; but the recevoirs enlarge towards the gland, and become smaller towards the nipple, which gives them a power of retention until the discharge of the milk is required. The milk next passes into the mammillary ducts or straight tubes of the nipple. These, like the recevoirs, are conical, with the apex of the cone turned towards the point of the nipple; and as their orifices at the nipple are very small and unyielding, the milk is also again retained until the act of sucking removes it; and when the draught occurs abundance of milk is hurried forwards to the recevoirs and mamillary tubes. The infant's lips and gums, and the suction produced by the exhaustion of the air in the mouth, not only mechanically empty the mamillary tubes and overcome the resistance of their contracted orifices, but also, by rendering them finer capillary tubes, assist, upon hydraulic principles, in giving rapidity to the passage of the milk." (p. 69.)

*The fat of the Breast.* Nature, says our author, has abundantly supplied this organ with adipose tissue, with the object—firstly, of preserving its general contour; secondly, of regulating its temperature under exposure; thirdly, of allowing it to float in an oily fluid, and thus of enabling it to elude or diminish the shock of external violence. This cushion of fat is placed beneath the skin, and is chiefly deposited in large lobes between the ligamenta suspensoria and the anterior folds of the glandular substance: it is not, however, secreted by these textures, but by a vascular membrane of the cellular adipose kind which lines them, and is not a simple investment, but gives off numerous processes, which by meeting and uniting form the little cellules that secrete and contain the smaller lobes of fat. Fat is similarly deposited posteriorly between the fascial loops connecting the breast with the aponeurosis of the pectoralis major muscle. The importance of this structure is sufficiently evident from various circumstances; for instance, from the impunity with which women of the lower orders receive severe blows in their pugilistic contests. From such encounters, says the author, I have seldom known them to suffer immediately any serious consequences; but he afterwards observes that "at distant periods women apply with tumours in their breasts, which they frequently impute to blows." When the period of lactation ceases, an increased quantity of fat is generally deposited, in order to supply the deficiency occasioned by the absorption of the glandular matter; but in very old age this also is absorbed, and the chest is then flattened like that of the male.

In the male subject but little fat is found connected with the mammary gland till an advanced period of life, when it is deposited not only in the folds of the suspensory ligament, anteriorly and posteriorly, but even in the interstices of the gland itself, and sometimes so abundantly as to render the breasts of aged men similar in appearance to those of females.

*Arteries.* It seems to be a matter of but little importance from what sources the glandules of the breast receive their supply of blood, since the distribution of the mammary arteries not only varies in accordance with the different positions of the gland in different animals, but is by no means constant in individuals of the same class. In the human subject the arterial blood is most commonly derived from the axillary and internal mammary arteries, the former usually sending off two posterior branches, the thoracica longa and the thoracica suprema, while the anterior or sternal arteries of the breast are generally three, viz.—the second, third, and fourth perforating branches of the internal mammary. The true external or posterior mammary artery is sometimes a branch of the thoracica longa, sometimes of the axillary artery itself. In addition to these vessels, the gland receives upon its costal surface one and sometimes two deep-seated branches from the mammary intercostals, which passing into the concave surface of the breast, freely supply its glandular structure. The branches of all these arteries, whether they are distributed to the external or internal structures of the breast, anastomose freely with each other. The arteries upon the cutaneous surface of the breast are lodged in the festoons formed by the ligamenta suspensoria; proceeding thence to the nipple, they send off parallel branches from its base to its apex, which again are minutely subdivided to supply the papillæ and ducts; they give off also some descending branches to anastomose with the arteries entering into the back of the gland.

*Veins.* "The branches of veins arising from the nipple pass from its papillæ in parallel branches to its base, and then form radii to an ellipse behind the areola at its margin. Their beautiful and minute division into branches upon the papillæ is seen in pl. x. figs. 3 and 4; and these, with corresponding divisions of the arteries, constitute the erectile tissues." From this ellipsis four principal branches proceed, and are distributed on the fore part of the breast in a freely communicating network. The chief cutaneous branches are more numerous than the corresponding branches of the arteries, and pursue a distinct course: the deeper-seated veins, however, and those of the interior of the gland, correspond in their course to the arteries of their respective regions. The mammary veins terminate variously in the axillary, cephalic, internal mammary and fourth mammary intercostal veins: a plexus also passes over the clavicle, and empties itself into the external jugular and subclavian veins. Such are their principal terminations, but they communicate also with the branches of the internal mammary veins, and "in a putrid body they colour the skin, and exhibit a beautiful and extended plexus, passing in all directions from the circumference of the breast." During lactation both sets of these vessels are serpentine in their course, as are also those of other parts of the body which are liable to changes of size, for instance, the uterus. Under the influence of certain malignant diseases the mammary veins become excessively distended with blood, and are the source of much pain to the patient and embarrassment to the functions of the chest. For the relief of this accumulation, the author has long been in the habit of opening these vessels with a double-edged lancet-shaped needle; and he thus, without exciting apprehension, can extract in three or four minutes from four to six ounces of blood, avoiding by this means the long-continued exposure of bleeding by leeches, and the trouble of continued fomentation afterwards.

*Nerves.* The nerves of the mamma are derived from the fourth, fifth, and sixth intercostals, and sometimes also from the third. Sir Astley describes those branches which enter it at its axillary margin as the posterior nerves, and those which penetrate the intercostal spaces between the cartilages of the ribs near the sternum, as the reflex branches. Their ultimate distribution, he says, can only be demonstrated by injecting the arteries upon which the nervous filaments are found lying as if for support. All the intercostal nerves derive filaments from the ganglia of the sympathetic; and to this circumstance the author attributes the relation which exists between the functional conditions of the mamma and uterus, in opposition to the opinion that the sympathy between the organs depends upon the anastomosis of the epigastric and internal mammary arteries. We quite agree with him here; for it is reasonable to suppose that during uterine gestation, when the uterine arteries receive an additional supply of blood for the development of the embryo, it is the internal iliac, and not the external, which is enlarged; as indeed is shown by the fact that an anæmial condition of the lower extremities is a frequent concomitant of pregnancy.

*Absorbents.* The absorbents are very numerous in the female breast, and are most easily demonstrated during lactation. They are divided into a superficial and deep-seated or cutaneous and glandular set: the former are situated immediately under the skin; the latter arise from the mucous membrane of the milk-cells and lactiferous tubes; and ramify, in the course of the veins, between the glandules and lobules of the breast. The superficial absorbents arise round the nipple; course upwards towards the axilla, lying first on the superficial fascia of the chest, which they afterwards pierce, and are then situated on the aponeurosis of the pectoral muscle. They continue this course upon the thorax as far as the third intercostal space; here they penetrate the fascia of the axilla, and forming a considerable plexus of vessels enter the axillary absorbent glands. These plexuses communicate with glands situated in the third intercostal space. They pass upwards to the second rib, surround the axillary vein, and, on reaching the first rib, again enter the absorbent glands. From the uppermost of these glands one large efferential trunk is formed, which runs up on the inner side of the axillary vein, and piercing the deep fascia of the neck between the clavicle and first rib, enters the vena innominata at the angle formed by the terminations of the internal jugular and subclavian veins.

All the superficial absorbents of the breast do not however take this course under the clavicle, for some may be traced running behind the axillary vein, artery, and nerves, and joining the absorbents of the arm, which then ramify through several glands, and again form plexuses which wind round the axillary nerves and vessels, and getting anterior to them above the clavicle, descend and terminate in the venæ innominatæ. If, therefore, says the author, the absorbent glands in the axilla become obstructed by disease of the breast, there are other vessels ready to perform their office, and should these be affected, the absorbents behind the scapula are capable of acting in their stead.

The absorbents on the inner or sternal side of the breast form themselves into an upper and lower plexus; the former, penetrating the intercostal space between the cartilages of the second and third ribs, enter the anterior mediastinum and pass into glands situated in the course of the internal mammary vessels, whilst the lower plexus enters the mediastinum, or between the cartilages of the fourth and fifth ribs, and unite with the others. These absorbents are described as uniting, on the right side, with some from the liver. On both sides they terminate in the venæ innominatæ. In pl. xi. figs. 1, 2, the course of the superficial absorbents of the mammæ is beautifully displayed.

No accurate description like the above of the superficial absorbents of the breast has been given by any preceding writer on the subject; for no anatomist has hitherto injected and dissected them so carefully as Sir Astley Cooper. Nor are these details of importance to the mere anatomist alone; they are fraught with the deepest physiological and pathological interest; but we must defer these considerations until we have described, after our author, the deep-seated absorbents.

The deep-seated absorbents are very numerous, as may be seen on referring to pl. xi. fig. 3. Arising from the internal mucous membrane of the cells and ducts, they ramify in the substance of the mammary gland, and a large proportion of them "unite to form two principal vessels, which pass into the axilla, and there enter the same absorbent glands which receive the superficial absorbent vessels." Those from the sternal aspect of the mamma pass into the anterior mediastinum, but some wind round the nipple and enter the axilla. The absorbent vessels of the concave surface of the breast take a different course: the posterior ones penetrate the intercostal muscles, and enter the absorbent vessels which accompany the aortic intercostal arteries, while the anterior, or those on the sternal side of the mamma, join the absorbents of the internal mammary arteries. The former pass into the thoracic duct in the posterior mediastinum, while the latter accompany the superficial vessels which have already been described. "A most extraordinary opinion has been broached," says our author, "that all the absorbents carry the chyle to the breast—an opinion at variance with the nature of the fluid, entirely inconsistent with every injection that I have made, as they all pass from and not towards the breast, and irreconcileable with the valvular structure of these vessels." (p. 88.) This paragraph well illustrates how easy it is for one who is in the constant habit of examining for himself by dissection the natural construction of the human body to overthrow an hypothesis founded upon no other data than those derived from a too fertile imagination.

To demonstrate to our readers the value of a thorough knowledge of the course of the absorbent vessels, we have only to refer to the history of malignant diseases which are propagated to distant organs by their means. Mainly on this knowledge must depend the power of forming a just prognosis, as well as of deciding whether or not an operation should be performed for the extirpation of the disease. The progress of malignant diseases in the course of the absorbents has never yet, we think, sufficiently occupied the attention of surgeons, either in this or in any other country; and we therefore consider this portion of Sir Astley's book as particularly valuable. This section concludes with a description of the course which malignant diseases of the breast follow in their propagation to the absorbent glands, and which the author points out as not being uniform; for when an affected gland becomes obstructed, the progress of the disease is checked in that immediate direction, and the poison will diverge into a collateral set of absorbents. "I have a preparation," he says, "which shows a plexus of vessels of communication at the roots of the absorbents, from which other vessels arise, taking a course into other glands; and thus when the glands in one axilla are obstructed, those of the other axilla will become similarly affected by absorbent spassing from the disease across the chest." (p. 88.) Sir Astley does not enter at all into the consideration of the particular malignant diseases of the breast, leading us to cherish a hope, which is strengthened by a passage in his Introduction, that he will shortly present us with a volume devoted entirely to that subject, in completion of the work which he has already commenced. From his pen we should doubtless receive, what is now very much wanted, a practical and precise exposition of the diagnostic marks by which, in the progress of malignant disease, the enlargement of the glands from the absorption of morbific matter, is to be distinguished from their increase in size resulting from common irritation; and we are sure that such an exposition, by such an author, would render the volume in question invaluable.

The structure of the mammary gland in the male, though the author dwells on it with considerable detail, appears to us to resemble, in most points, so nearly that of the female, that we shall proceed at once to the most peculiar and apparently most important of its constituents—its absorbent glands. With regard to its general organization, however, we will just observe that it consists of two parts—of very minute cells, and of small conical ducts, which divide into numerous branches in the gland, and terminate in straight ducts, which end in very minute orifices in the nipple. In their form, divisions, and course through the nipple, they all form a miniature resemblance of the gland in the female. The most extraordinary part of the structure of the male breast is its absorbent vessels, which are comparatively larger and more numerous than in the female, though we are not acquainted with any use which can be attributed to them. Sir Astley describes, at length, their course and the situation of their glands, with frequent reference to the plates 2 and 3 of the male breast, in which they are delineated. With respect to their function, he concludes with the following remark:

"It appears from this account of the absorbent vessels of the male breast, that when any secretion proceeds in it, as there would be great difficulty in its escape at the small orifices at the nipple, the fluid is taken up by the absorbent vessels, and carried into the circulation. Whether this fluid is necessary or not to the blood, I have had no opportunity of ascertaining, but the structure is very curious, and the assemblage of absorbents is quite extraordinary." (p. 186.)

There is nothing more remarkable in the relative conformation of the sexes—particularly when we consider their respective distinguishing characteristics—than the structural identity of their mammary apparatus, which in both is at such an advanced stage of development at the period of birth, as seems to prove that it performs some important function in the animal economy, unconnected with the generative organs. It would be absurd to suppose, as has been stated, that the breast of the male exists only for the sake of symmetry, inasmuch as it is comparatively larger at birth than at any subsequent period, and produces then, like that of the female, a secretion. This identity continues until puberty, when a new action is set up in the female breast, rendering it directly and uniformly sympathetic with the organs of generation, and preparing it to furnish nourishment to the young. In the unimpregnated female it is probable that a secretion is constantly going on, which is regularly conveyed into the venous blood. And even at parturition the quantity of milk secreted is so much greater than is required for the support of the infant, that a considerable portion is re-absorbed into the blood of the mother, and serves again for her nourishment. At the epoch of puberty in the male, when the generative organs suddenly increase, and seminal secretion commences, the mammary glands always become excitable and undergo a certain change, showing that they sympathize, at any rate to some extent, with the organs of generation. Is it possible that they furnish any elements to the blood, or cause it to undergo a change, fitting it for the elimination of the seminal secretion? In the female, as soon as lactation ceases, the breast reverts to the size and conditions which it presented previously to conception. During menstruation it invariably becomes tumid, apparently from an increased determination of blood. Is it the purpose of this process that albumen, in some form or other, may be secreted in the breast and be conveyed into the general circulation to compensate for the loss sustained by the menstrual excretion? At the period of life when menstruation ceases, the glandular portion of the breast diminishes nearly to its size before puberty. This diminution, however, is not always obvious, as the deposition of fat, in many instances, causes the breast to retain its former external appearance.

*Evolution of the Breast.* In the section of this work, devoted to the evolution of the breast, the author has accurately described the progress of development of the various constituent structures of the mamma. In the fœtal state, the mammary gland is found immediately behind the nipple, is of a rounded form, and imbedded in the subcutaneous adipose tissue. Its vascularity distinguishes it at once from the surrounding parts, and it presents indeed a well-defined circumscribed body. But slight indication of a nipple is yet to be perceived; and this part is rather a cleft than a prominence. At birth a thick secretion may be pressed out of the excretory ducts, of which circumstance nurses are well aware, and are too prone to exercise undue pressure in removing it, from an erroneous idea that its presence is injurious. The same appearances are presented at this period by the glands of the female and male, as will be seen in comparing pl. ii. of the former with pl. i. of the latter. During the first year of life, the gland diminishes both in size and vascularity. Between the second and third years it becomes separated and even isolated as a distinct organ, by being inclosed, as has been above described, in the fascia mammæ. It undergoes but little further alteration till the ninth year, except that its figure becomes less rounded. (See figs. 1, 2, 3, 4 of pl. ii.) At the age of eleven and twelve, the diameter of the gland is greatly and suddenly increased. At thirteen, its anterior surface becomes concave, whilst its edges are turned up, from the breast growing faster than the fascia mammæ. At fourteen, there is a perceptible increase in size. At sixteen, not only has its evolution greatly advanced, but, according to our author, some of its lactiferous tubes can be injected. At from twenty to twenty-one, the gland has obtained its full size before lactation, and all its constituent structures are perfected. (See fig. 5 to 11, inclusive, of pl. ii.)

Soon after birth the cleft which had occupied the position of the nipple becomes converted into a cone, and an areola appears around it, which increases but little in size up to the tenth year, when it somewhat enlarges. At twelve, not only are the nipple and areola increased in size, but small glands are perceptible on the surface and at the circumference of the latter. At fourteen, the nipple is still further increased; its papillæ begin to evolve, with small clefts between them; the areola becomes convex from the growth of the mammary gland behind it, and both it and the nipple are of a bright red hue. The roundness and prominence or intumescence of the breasts now appear. From fifteen to seventeen, earlier or later, according to the temperament of the individual, the nipple becomes perfectly evolved; the areola is now more than an inch in diameter, and its tubercles are enlarged. No further change is perceived prior to lactation. It is singular that the areola is not present before birth, either from such a congeries of vessels not being then required, or from the dark colour of the blood not becoming perceptible until it has been exposed to the atmosphere.

*Influence of Gestation.* The next section of the work treats of the effects of gestation and lactation upon the breast. During gestation the mammæ receive much larger quantities of blood than usual: they generally swell, become painful, feel heavy and are tender to the touch: if small before, they now undergo their proper evolution. The nipple increases in size, and its papillæ and mucous follicles are developed. The areola becomes darker in colour, thicker, and its diameter increases from one to two inches; the rete mucosum being much enlarged. We may add to these summary signs of pregnancy the not infrequent occurrence of a secretion and discharge of milky serum, although sometimes there is no appearance of milk until after parturition. The above phenomena are not infallible signs of pregnancy, as they may also accompany hypertrophy, polypus, and some other affections of the uterus. In cases of uterine disease, however, it may be remarked, the areola is neither so dark nor so much developed as in pregnancy; and this circumstance should be borne in mind as constituting an important distinction between real pregnancy and morbid affections which simulate it. It is not perhaps impossible that during pregnancy the external atmosphere may act upon the blood in the areola, and thus influence in some way the secretion of milk. The tumid enlarged state of the mammæ continues until after parturition; but it is not until after suckling has gone on for some time that the milk-cells become fully developed; so that a woman, says our author, who dies of puerperal fever is not the best subject from whom to take the mammæ for the purpose of injection. After a few weeks' suckling the nipple becomes much elongated, its papillæ prominent, and its extremity enlarged, so as to facilitate the adhesion of the infant's lips.

*The secretion of milk* commences generally on the third or fourth day after the birth of the child. Directly after parturition the blood, which is no longer required by the uterus, is directed to the breast for the secretion of nutriment for the infant. The author states that excitement, both constitutional and local, attends the commencement of lactation, a sufficient proof that the mother is urged by nature to suckle her infant as soon as her breasts can supply it with milk; for immediately the child partakes of the proffered nourishment, the painful tension of the breast subsides, and all the symptoms of irritative fever are relieved. The action of the child's lips is the natural means of relieving the breast and of causing the secretion of milk to continue; the first fluid, however, which the infant extracts is not milk, but is a secretion believed to be of essential service as a purgative in removing the meconium which might otherwise accumulate and become injurious. The early application of the child to the breast has also the advantage of elongating the nipple and fitting it for its future functions, before the glands have become too distended and hardened by the abundance of the secretion of milk. After lactation is established, the secretion of milk is either constant or occasional. In the former case the milk-tubes are supplied by a slow and continued production of fluid, which being retained in them for a long time undergoes a certain change, rendering it fitter for the nourishment of the child. Such retention is evidently requisite, for when the child is too frequently put to the breast, the milk evidently loses some of its nutrient qualities and the mother is also debilitated. The occasional secretion is that which is termed by mothers and nurses *the draught*, when there is a sudden rush of blood to the gland, and the milk is secreted so suddenly that it sometimes spirts out of the nipple. The sight of the child or the pressure of its hand or head to the breast are the most frequent causes of occasional secretion. It has been supposed by some physiologists that when the breast is from any cause distended with milk, the latter passes into the surrounding cellular membrane and absorbent glands; and Haller has spoken of the vessels which convey it to the latter. It was this doctrine, perhaps, which led to the absurd hypothesis that the constituents of the milk are furnished by the absorbents: a supposition which seemed to be favoured by the fact that the mammary absorbents are eight times more numerous than the blood-vessels; and also because the milk more resembles chyle than blood, both the former fluids containing muriate of potash, of which there is none in the latter.

The supply of milk in the human female is often superabundant, and has to be removed by artificial means; neither its quantity nor its quality, however, seems to depend upon the size of the mammæ, but rather upon the constitution and general health of the individual. Sir Astley found that the quantity of milk furnished by the breast of a woman whose infant was eighteen weeks old was two ounces, but only half that quantity when it was drawn off more than once a day. A woman seventeen months after delivery milked two ounces from her breast when the child had been seven hours absent from her. The morning's milk he has almost constantly found greater in quantity than the evening's. The secretion of milk will continue for many years in a healthy mother, if encouraged by the application of the child to the breast; and the author cites instances of the great length of time it has endured, and even of mothers having suckled two of their children consecutively without any intermission of the secretion. Wet nurses have also been known to suckle two children consecutively. Sir Astley considers that the proper time for weaning is sufficiently indicated by the appearance of teeth, and by the concomitant capacity of the child to digest other food. The secretion, should it continue after this period, may be suppressed by the application of evaporating lotions and the administration of active purgatives in the morning.

After mentioning the causes, such as inflammation, the formation of abscess, general constitutional derangement, &c., which prevent a mother from suckling her infant, the author observes that this duty is too often neglected from caprice, the fear of trouble, the dread of spoiling the figure, or from anxiety to avoid the confinement it requires; and in some cases it may arise from a desire to bear a great number of children. Women who become pregnant again during lactation should wean the child, as the milk is now deteriorated in quality and disagreeable. This change may be easily explained when we reflect that the new function of the uterus must necessarily interfere with the supply of blood to the breasts. Should the mother be compelled from any cause to wean her child prematurely, there is no necessity, according to Sir Astley, and in this view he is supported by Drs. Walshman and Key, that the wet nurse who succeeds her should have been giving suck for precisely the same period; inasmuch as after the first few weeks of lactation the milk does not undergo any appreciable change. It is a singular fact that should the child be deprived of the breast from any cause and should then be put to it again after the lapse of several weeks, the secretion of the gland will return and the child be supported by it.

The advantages which accrue to both mother and offspring from lactation are beautifully illustrated by the author. After remarking that women who have been previously delicate become strong and healthy whilst they suckle, he describes other numerous benefits which they derive from the performance of this duty.

"The giving suck may be the means of preventing or of lessening the tendency to puerperal fever by determining the blood to the breast for the secretion of milk, and withdrawing it from the uterus, peritoneum, and iliac vessels...... Suckling also diminishes the disposition to malignant diseases of the breast; for although women who have had children are still liable to cancerous and fungoid diseases, yet it is undoubtedly true that breasts which have been unemployed in suckling, in women who have been married but who are childless, and in those who have remained single, are more obnoxious to malignant diseases than those of women who have nursed large families; and if it were only to lessen the probability of the occurrence of such horrible complaints and causes of dissolution, women ought not to refuse suckling their offspring. A woman who has children and has suckled them is undoubtedly a better insurable life than a married woman who has no children or one who has remained single. A female of luxury and refinement is often in this respect a worse mother than the inhabitant of the meanest hovel, who nurses her children and brings them up healthy under privations and bodily exertions to obtain subsistence, which might almost excuse her refusal. The frequent sight of the child, watching it at the breast, the repeated calls for attention, the dawn of each attack of disease, and the causes of its little cries, are continually begetting feelings of affection which a mother who does not suckle seldom feels in an equal degree, when she allows the care of her child to devolve upon another, and suffers her maternal feelings to give place to indolence or caprice, or the empty calls of a fashionable and luxurious life. It is, however, melancholy to reflect that a life of high civilization and refinement renders the female less able to bear the shocks of parturition, less attentive to her offspring, and less capable of affording it its natural nourishment; so that she is often a worse mother in these respects than the female of the middle ranks of life or even the poorest cottager." (pp. 137-8.)

Besides the other evident benefits which the child derives from the breast of its mother, this is its natural pillow, and affords it especial relief during the irritation attendant on dentition, when, says Sir Astley, "the mother's anxiety contributes to the relief of the child, for it renders her milk a purgative, and thus acts usefully as an aperient when the system is in a feverish state, and thus operates as the best medicine." When, however, the mother is from any cause incapable of nourishing her offspring, the application of the child to the breast of another is infinitely preferable to bringing it up by hand. Dr. Merriman is of opinion that taking into consideration all classes of society, not more than two in ten children nourished by the latter method survive from eighteen to twenty months.

With respect to the appropriate food of the mother during lactation, the author, who has investigated this subject at considerable length, and has received information from different parts of the country on the habits of women who nurse their own children, has arrived at the conclusion that in the higher classes of society, at any rate, women take food in greater quantity and of a more stimulating nature whilst suckling than is either necessary or advantageous. Still no general law can be laid down either as to the quantity or quality of the food to be taken; and the instinctive cravings of the mother will be found the best indication of the amount of nourishment required. Improper diet of the mother, for instance fruits in some cases, or acids and fermented liquors, may injure the quality of the milk; which may also be deteriorated by the return of the menstrual secretion, on the appearance of which weaning should instantly be recommended, as the mother is incapable of supporting the double secretion, and as the milk is now rendered unfit for the support of the infant. In reference to the influence of various mental conditions upon the milk, the author confirms, from his own experience, the injurious effect of fits of anger, grief, anxiety, &c. upon its secretion, and narrates two cases in which females in consequence of a sudden alarm were compelled to give up suckling.

The milk is capable of being greatly changed in quality by pharmaceutical agents. This is proved by this circumstance amongst many others, that numerous children in our hospitals with a congenital venereal taint are cured by the administration of mercury to the mother. Purgatives very readily affect the milk, and act through it upon the bowels of the child. Olive oil, castor oil, confection of senna, and the compound extract of colocynth, Sir Astley seems to regard as the best purgatives for women during lactation: saline cathartics he disapproves of as likely to produce too violent an effect upon the infant at the breast. The milk of a patient of Guy's Hospital who had been taking iodine for a fortnight was tested by Dr. Rees with sulphuric acid and starch, and strong indications of the presence of that medicine were thus detected. Chevalier, Henry, and Peligot administered various medicines to asses, and found traces of them afterwards in the lacteal secretion; of common salts, for instance, in abundance, as also of sesqui-carbonate of soda, and of sulphate of soda when administered in doses of two ounces. Iodide of potassium also affected the milk, as did the oxide of zinc, the tris-nitrate of bismuth, and sesquioxide of iron; but sulphate of quinine, the alkaline sulphurets, the salts of mercury, the nitrate of potass, could not be detected, even after the ingestion of these drugs in considerable doses.

For some interesting particulars respecting lactation amongst the blacks in the West Indies, contained in two letters addressed by Dr. Young to Sir Astley, we must refer the reader to the work itself.

*Milk.* The author enters at some length into the subject of the chemical composition of the milk; but we can only find room for a few of the more interesting particulars. He first describes the qualities and proximate principles of this fluid in the mammalia generally. He observes that its component parts unite the qualities of animal and vegetable matter, and are diluted and combined by a watery solvent. Its colour depends upon a number of oily globules, which float through the fluid, forming an opaque emulsion with the caseous matter, and which may be separated by filtration with good blotting paper, the remaining fluid being thus rendered clear and transparent. These globules not being chemically combined with the other constituents rise to the surface of the milk, when it is allowed to rest and form the layer of cream.

"Human milk when first drawn appears more blue in its colour than that of the cow; indeed it resembles whey, or cow's milk much diluted with water." Soon after it is drawn, if allowed to rest, it divides like ordinary milk into cream and milk, "but with this striking difference that the milk in the human subject appears semi-transparent like whey, instead of being white and opaque like that of the cow, so that it may be said to divide into cream and whey." During the first ten days of its remaining at rest, abundance of cream and a little curd are separated; lactic acid is also developed; but it does not appear from the experiments of the author that these changes are completed until the milk has been exposed for a period of two months. According to Dr. Rees its specific gravity appears to be 1·0358: its solid contents 12 per cent. The specific gravity of the cream is 1·021. Widely different from what is observed in other animals, the cream in the human milk varies in quantity according to the health, food, habits of life, and temperament of the mother, and also according to the age of the child. From a series of experiments detailed in the work, it would appear that the quantity of cream is from one fifth to one third that of the milk, the variation depending upon the causes above mentioned. "In a woman who was very poor, and who had an exfoliation of the os frontis, seven measures of milk gave only one of cream, while in a woman highly fed, nine measures of milk had three and a half of cream." In the same individual the quantity varies according to the time which has elapsed since the birth of the child. From Sir Astley's experiments, the results of which are embodied in a table, it seems established that during the first eight days a very large proportion of cream is formed; the variations which occur subsequently appear inexplicable in the present state of our knowledge.

This section of the work concludes by a communication on the nature and chemical composition of milk from the pen of Dr. Golding Bird, which contains an excellent summary of our present knowledge, and which we recommend to the attention of our chemical readers.

*Comparative Anatomy.* In the chapter on the comparative anatomy of the mammary gland, which concludes the present work, that structure is described as it exists in some species of the classes of graminivora, carnivora, and omnivora. Its secretory apparatus in all mammalia bears a strong resemblance to that in the human female and presents the same constituents. In all there is a prominent nipple, with the exception of the whale tribe and the ornithorynchus. In the number of the mamillary tubes or ducts, however, considerable varieties are found. The cow, the ewe, and the goat have but one tube to each teat, the rhinoceros, on the other hand, has twelve, probably from the great size of the young rhinoceros requiring a freer exit for the milk. The pig has two tubes in each teat; the hare, rabbit, cat, and bitch have several; the guinea-pig and porpoise only one. The graminivora differ from the carnivora in the size of their reservoirs, which are enormously large. The supply of blood, as has been before observed, varies in its source according to the position of the gland in different animals, but it is nevertheless equally available in all cases for the purposes of secretion. The nerves, too, distribute the same influence, and are similarly furnished with anterior and posterior filaments from the medulla spinalis, as well as a branch from the sympathetic, whether they are derived from the cervical, dorsal, or lumbar regions of the spinal marrow. It is a singular fact that the mammary glands are the only secretory organs which derive their nerves principally from the spinal cord.—This chapter is illustrated by ten plates, of several of which the descriptive text contains an analysis of the milk of the respective animals.

We have now laid before our readers not only by far the most complete account of the anatomy of the breast ever published, but also one which leaves little if anything more to be done on the subject by future enquirers. Sir Astley Cooper will always be the standard authority on the structure of the mammæ, and the work we have reviewed will be as valuable to our successors as it is to us, the contemporaries of its author. We venture again to hope that it will shortly be followed by a volume on the malignant diseases of the breast.

In taking leave of our author, and in thanking him warmly for the good service he has now and ever done to science, we need hardly wish him any fresh reward for his toils; for a life spent as his has been, is, and we doubt not will be, in the successful pursuit of truths the most important to the well-being of mankind, brings with it infallibly the loftiest and purest enjoyment: but we will pray divine benevolence to grant that one who has conferred so many blessings on his race may yet long be spared to enjoy the gratitude and honours which he has so nobly acquired.

